# Study of polarized activated carbon filters as simultaneous adsorbent and 3D-type electrode materials for electro-Fenton reactors

**DOI:** 10.1016/j.jece.2020.104414

**Published:** 2020-10

**Authors:** Irma Robles, Gabriel Moreno-Rubio, Josué D. García-Espinoza, Carolina Martínez-Sánchez, A. Rodríguez, Yunny Meas-Vong, Francisco J. Rodríguez-Valadez, Luis A. Godínez

**Affiliations:** a Centro de Investigación y Desarrollo Tecnológico en Electroquímica, Parque Tecnológico Querétaro Sanfandila, 76703, Pedro Escobedo, Querétaro, Mexico; b CONACYT – Centro de Investigación y Desarrollo Tecnológico en Electroquímica, Querétaro, Mexico

**Keywords:** Activated carbon, 3D electrode, Electro-Fenton, Advanced oxidation processes

## Abstract

Electro-Fenton (EF) based water treatment processes using activated carbon (AC) packed beds constitute an attractive approach for the development of competitive degradation technology of persistent pollutants in aqueous effluents. In this work, the results of a study aimed to assess the effect on the EF performance of different parameters of the reactor's operation are presented. By means of a factorial experimental design, the influence of the AC source (lignitic or vegetal), AC acid pre-treatment, particle size distribution and the amount of Fe loaded resin in the reactor were analyzed. From the resulting data it was found that the most influential parameter in the EF performance of the reactor is the AC source. Modest effects were observed for AC acid pre-treatment, which limits Fe ion adsorption on the AC substrate. The use of a wide particle distribution of AC particles was also found to improve inter-particle electrical contact, thus favoring the electrochemical processes that take place inside the reactor. An investigation on the effect of the amount of Fe in the reactor as well as its distribution dynamics, also revealed that an excess of Fe ions in the reactor decreases the EF performance of the system since Fe ions efficiently adsorb on the AC substrate, particularly in non- acid treated samples. The best operation conditions consisted on using un-meshed vegetable AC, without acid pretreatment in an EF reactor loaded with 0.25 g of Fe, which allowed to reach full color removal of bright blue FCP model dye in 70 min.

## Highlights

• An Activated Carbon (AC) bed simultaneously works as adsorbent and electrode.
• A DOE was used to study the effect of AC on the performance of an EF reactor.
• The effect of AC source was found to be the dominant factor on EF performance.
• AC mesh separation and acid pre-treatment have modest effects.
• Excessive amounts of the Fe results in limited performance of the EF reactor.

## Graphical abstract

**Figure:**
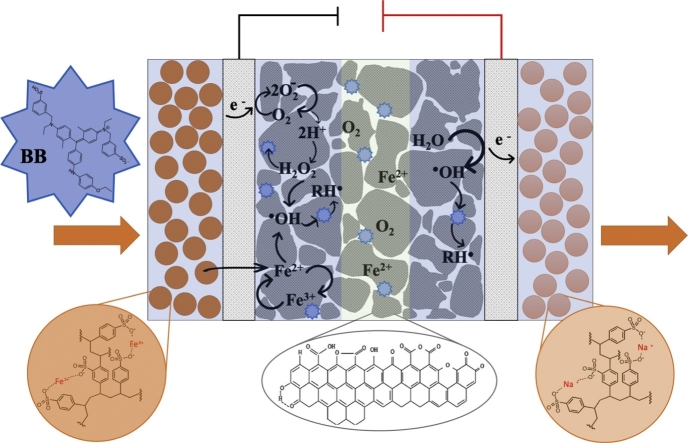

## 1 Introduction

Activated carbon is by far the most popular adsorbent for water treatment processes. It is a relatively cheap and available material with high surface/mass ratio that is widely used in domestic, laboratory and industrial processes. According to Garside (2020), it is expected that by 2021, the market volume of activated carbon will reach 3.31 million metric tons worldwide [1] and therefore, the associated challenge for its efficient regeneration and disposal is the subject of research of several environment focused groups around the world.

Carbon materials can be obtained from mineral extraction [2] or from biomass sources such as animal feedings [3], agricultural [4] or woody materials [5], food wastes [6] and sewage sludge [7,8], among others [9].

These source materials are activated either with HNO_3_, H_2_SO_4_, HCl, H_3_PO_4_, NaOH or KOH and then thermally treated by pyrolysis, carbonization or hydrothermal treatment, to obtain activated carbon (AC). In addition to their excellent adsorbent properties, AC substrates are characterized by reasonable electric conductivity and a high density of polar, oxygenated surface chemical units such as carboxylic (—COOH), hydroxide (-OH) and carbonyl (-CO-) functional groups [10,11].

In the context of the wide variety of combinations that can be anticipated for AC preparation due to different sources, thermal treatments and activation steps, there is a large number of AC substrates characterized by different physical and chemical properties that make them suitable as adsorbent materials for a variety of adsorption processes. It is also important to note that although AC materials are usually employed in purification technologies, some recent studies have explored their use in catalysis [[12], [13], [14]] as well as in Advanced Oxidation Processes (AOPs) [[15], [16], [17], [18], [19]].

AOPs are pollutant degradation processes of aqueous effluents characterized by the use of the hydroxyl radical species (^•^OH) as the main oxidizing agent. The hydroxyl radical is extremely powerful (*E°*_(_O^•^_H/H2O)_ = 2.8 V *vs* SHE) and as pointed out by Sirés et al. (2014), it is capable of mineralizing contaminant molecules *via* hydroxylation or dehydrogenation reactions [20]. In addition, the ^•^OH radical species is non-selective and as opposed to halogens, gives rise to hydroxylated non-toxic by-products. Since ^•^OH radicals are highly reactive, their half-lifetime is also quite short and therefore they cannot be produced, stored and transported for later use in a water-treatment process. Instead, ^•^OH radicals must be prepared *in-situ* and the different approaches for their production and use, define the different AOPs.

Among the AOPs, the Fenton mixture is with no doubt one of the most popular approaches. The Fenton reagent consists on the mixture of Fe^2+^ and H_2_O_2_ which, as can be seen in Eq. (1), results in the production of the ^•^OH radical species.

(1) $${\text{F}\text{e}}^{2+}+{\text{H}}_{2}{\text{O}}_{2}\to {\text{F}\text{e}}^{3+}+\;{\;}^{•}\text{O}\text{H}+{\text{O}\text{H}}^{-}$$

Since H_2_O_2_ solutions are expensive, difficult to handle and chemically unstable, an interesting and potentially important approach consists on the electro-Fenton (EF) process. As can be seen in Eq. (2), in this electrochemical AOP, H_2_O_2_ is electrochemically produced by means of the 2e^−^ oxygen reduction that, under slightly acidic conditions, takes place on the surface of carbonaceous electrodes,

(2) $${\text{O}}_{2}+2{\text{H}}^{+}+2{\text{e}}^{-}\to {\text{H}}_{2}{\text{O}}_{2}$$

The main disadvantages of the EF process however, consist on the need to acidify and add the Fe(II) ions to the influent before the degradation process takes place and the requirement of neutralizing and removing the iron ions from the treated aqueous solution. As expected, the corresponding costs associated to these operations have proven to be serious limitations for the development of EF based wastewater treatment technologies.

**Fig. 1:**
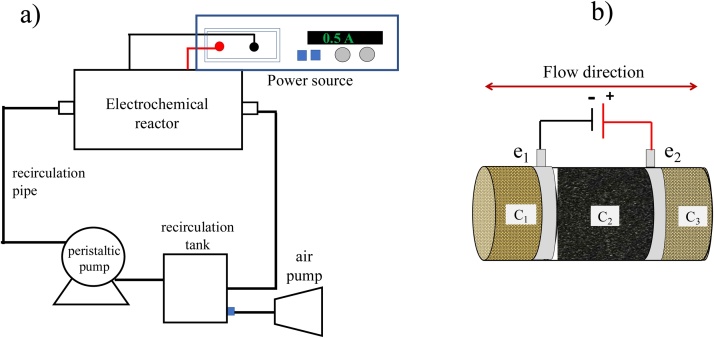
Schematic diagram of the a) experimental set-up, b) the electro-Fenton rector.

Recent studies by several research groups around the world however, have suggested novel arrangements and reactor designs aimed to overcome these limitations. Our group for instance, has reported studies on a reactor consisting on three compartments connected in series for which cation exchange resins loaded with Fe(II) and H^+^ are used to provide and remove these cationic species, to and from, an EF reactor (see Fig. 1b). While the cation exchange resins are positioned in the first and third compartments of the reactor delivering and retaining Fe and acid during a *t_1_* period of time, the central section is loaded with a polarized AC bed that simultaneously acts as an adsorbent and as an EF reactor where degradation of the pollutants takes place. As can be seen in Fig. 1 and Table 1, this reactor arrangement is capable of working in sequential cycles for which polarization and influent flow direction are simultaneously switched at a fixed frequency (1/*t_1_*) so that the Fe and acid species do not leave the reactor; thus overcoming the need to add and to remove these cationic species to the EF wastewater treatment reactor.

**Table 1:** Operation conditions for the sequential adsorption/polarization stages.

Cycle	Stage	Time (min)	Accumulated time (min)	Flow Direction	Polarization (e_1_ - e_2_)
Ads	Adsorption	20	20	→	
1	Polarization	10	30		(-) (+)
	Adsorption	15	45	→	
2	Polarization	10	55		(-) (+)
	Adsorption	15	70	⟵	
3	Polarization	10	80		(+) (-)
	Adsorption	15	95	→	
4	Polarization	10	105		(-) (+)
	Adsorption	15	120	⟵	

From EF studies using this arrangement, we have showed that it is possible to effectively treat aqueous effluents contaminated with dyes [21], recalcitrant contaminants [22] and the full inactivation of pathogens such as *E. Coli* and Helminth Ova [23]. We have also reported some important aspects of the characteristics that define the performance of the AC in this reactor and although we have explored the adsorption regeneration properties, the cathodic protection [24,25] and the influence of the carbon particle density on the electrical conductivity of the AC packed bed compartment [[21], [22], [23],^26^], there are still some important questions such as the influence of the AC source, size and pre-treatment process before the AC material is used in the EF treatment reactor. It is in this context that we present a report on the results of a study in which we assess the effect that some of these AC related variables exert on the performance of the EF reactor shown in Fig. 1.

## 2 Experimental

### 2.1 Materials

Chemicals employed for electrolyte preparation consisted on analytical grade Na_2_SO_4_, FeSO_4_7^•^H_2_O, H_2_SO_4_, HCl and HNO_3_ that were obtained from *J.T. Baker* and used as received. For dye degradation studies, 100 mg L^−1^ solutions of bright blue FCP (CAS: 3844-45-9) were employed using Na_2_SO_4_ at pH 3 as solvent.

While analytical grade coumarin was purchased from *Sigma-Aldrich*, CG8-C cation exchange resin and carbon cloths were obtained from *Resintech* (Mexico), and *ROOE* (Mexico), respectively. The resins and the carbon cloths were cleaned and pre-treated as previously described by Zárate et al. (2018), and García-Rodríguez, et al. (2017) [27,28]. The supporting electrolyte consisted on pH 3 Na_2_SO_4_ (0.05 M) dissolved in deionized water (DIW, 18 MΩ cm).

Activated carbon samples, vegetal (VAC) and lignitic (LAC), were both obtained from *Clarimex* (Mexico). According to information provided by the supplier, while vegetal AC was prepared from wood VG 14 × 35 featuring a surface area of 1091 m^2^ g^−1^, pore volume of 1.11 cm^3^ g^−1^ and pore size of 4.07 nm, mineral or LAC samples were produced from lignite CAGR 8 × 30 with a surface area of 548 m^2^ g^−1^, pore volume of 0.57 cm^3^/g, and pore size of 4.17 nm.

AC materials were either washed with ultra-pure deionized water (DIW, 18 MΩ cm), or pre-treated with sequential washing steps using HCl and HNO_3_ solutions (0.01 M and 10 % v/v respectively), under constant stirring for 2 h. After acid pre-treatment, the AC samples were thoroughly rinsed with DIW until a neutral pH value was measured in the washing effluent and later, the AC samples were dried overnight in an oven at 60 °C.

AC packed beds were prepared using different particle size (*x*) distributions. In this way, mesh separation of VAC and LAC was carried out using the following mesh sizes and sample keys. For VAC, the interval of small particle size (Low level (-)) was: 0.85 ≤ *x* ≤ 1 mm and the interval for medium particle size (Medium level (0)) was 1.0 ≤ *x* ≤ 1.18 mm. For LAC, the interval of small particle size (-) was: 1.4 ≤ *x* ≤ 1.7 mm and the interval for medium particle size (0) corresponded to 2.0 ≤ *x* ≤ 2.36 mm. AC Samples to which no mesh size separation was applied, were identified with the High level (+).

### 2.2 Electrochemical experiments

Electrochemical experiments were carried out using a 145 mL polymethacrylate reactor coupled to a power source (*PS, Novak Technologies*), a peristaltic (*Masterflex*), an air (*Elite 799*) pump, a recirculation tank equipped with the corresponding tubing, flowmeter and connectors (see Fig. 1a). As described by Fernández et al. (2018) and García Espinoza et al. (2019) [21,22], the reactor consists of three cylindrical pieces interconnected in series using stainless steel screws (see Fig. 1b). While the central compartment (C_2_) contains a packed bed of polarized AC (of either vegetal or mineral origin) positioned between two graphite clothes that worked as electrical contacts for polarization purposes (e_1_ and e_2_ with a geometric surface area of 4.91 cm^2^ and an inter-electrode distance of 6.5 cm), compartments C_1_ and C_3_ were filled up with cation exchange resins. While the compartment adjacent to the cathodic side of C_2_ was loaded with Fe(II) and acid as has been previously described [21,22], the other compartment, positioned on the anodic side, contained resin previously exchanged with Na^+^ cations. The volume of solution treated in electrochemical experiments using this reactor was 250 mL which were pumped in continuous recirculation mode at a flow rate of 30 mL min^−1^ under a cyclic scheme (see Table 1) in which adsorption and polarization sequential stages take place [21,22]. During the polarization stage, 0.5 A (corresponding to about 8 V) were applied between e_1_ and e_2_ using a power source. The electrolytic solution on the other hand, was maintained under oxygen saturation conditions by means of continuous bubbling and stirring in the recirculation tank, as schematically shown in Fig. 1a.

Color removal percentage (CR%), was assessed by measuring the absorbance decrease of the model dye solution at 629 nm using Eq. (3) where A*_0_* and A*_t_*, correspond to the Absorbance values at the beginning of the experiment and at time *t*, respectively. Total Fe concentration on the other hand, was measured following the APHA methodology. Specifically, we employed the 3500-Fe D technique which is based on the reaction of iron with phenanthroline chelate to form an orange-red complex. Since the generated colored solution follows Beer's law, the concentration of iron could be determined using a spectrophotometric absorption measurement at 510 nm. A calibration curve of known Fe concentration *vs* absorbance was used to determine the residual Fe concentration in solution [29]. While the spectrophotometric tests were carried out using a *Genesys* 10S, *Thermo Scientific* equipment and fluorescence measurements were performed utilizing a Cary Elipse, *Agilent* apparatus, pH and electric conductivity were measured employing a *Thermo Scientific* potentiometer.

(3) $$\text{C}\text{R}\;\left(\text{\%}\right)=\frac{A_{0}-A_{t}}{A_{0}}\;100$$

Since the goal of this work was to study the influence of some AC variables that characterize the performance of the EF system shown in Fig. 1a, a multilevel design of experiments (DOE) approach was selected to carry out the study. This statistical approach is widely used not only to explore the influence of experimental parameters in multi-variant systems, but more importantly, to assess the effect of coupled-variable effects. In this way, experiments were carried out in random order and performed in duplicate. For the analysis of the data resulting from these experiments, the software Statgraphics *Centurion XVII*, was employed.

The analysis of variance (ANOVA) allows to identify the effects of different variables and their individual and combined effects on the response variable. A P-*value* lower than 0.05 indicates that the variable and its interaction is significant for the process assuming a confidence level larger than 95 %. The ANOVA is strongly related to the Pareto diagram, which is commonly applied to test the null hypothesis that refers to color removal. Since the standardized Pareto diagram plots the estimates of standardized effects, the critical value is obtained from reference numbers for the Student's *t*-distribution. In this way, if the absolute value of the standardized effect is larger than the critical value, then the corresponding population effect will be significantly different from zero.

## 3 Results and discussion

### 3.1 Determination of the polarization time for the EF process. Detection of ^•^OH radical production

As it was previously pointed out, the goal of this work was to study some of the variables involved in the preparation of the AC packed bed that in an EF reactor simultaneously works as an adsorption material (filter) and as a 3D-type electrode substrate. In order to do so, it was first necessary to determine the operation conditions (adsorption and polarization sequential steps) that needed to be fixed for the reactor’s operation so that in the following stage, the variables of the AC filter could be properly defined to work out the DOE and then, determine the effects under study employing the statistical analysis of the experimental results.

Using the reactor shown in Fig. 1 and the information reported by Fernández et al. (2018), García-Espinoza et al. (2019) and Robles et al. (2020), a potential difference of 8 V (corresponding to 0.5 A), a concentration of Fe(II) of 2 mM and a flow rate of 30 mL min^−1^, were selected to find out the polarization period of time that maximized the concentration of ^•^OH produced from the Fenton mixture (Fe(II) and H_2_O_2_ that results from O_2_ reduction at the cathodic surface) [[21], [22], [23]]. Since the ^•^OH lifetime is rather short (approximately 40 μs [30]), indirect methods based on scavengers have been developed for its quantification. These methods include spin-trapping techniques such as electron paramagnetic resonance [31], chemiluminescence [32], liquid chromatography [33], spectrophotometry [34] and fluorescence [35]. In this regard, coumarin is one of the most popular trapping compounds, which reacts directly with ^•^OH to produce the fluorescent 7-hydroxycoumarin (7—HC). The amount of 7H—C generated in the reaction systems could be related with the amount of produced ^•^OH [[35], [36], [37], [38]].

**Fig. 2:**
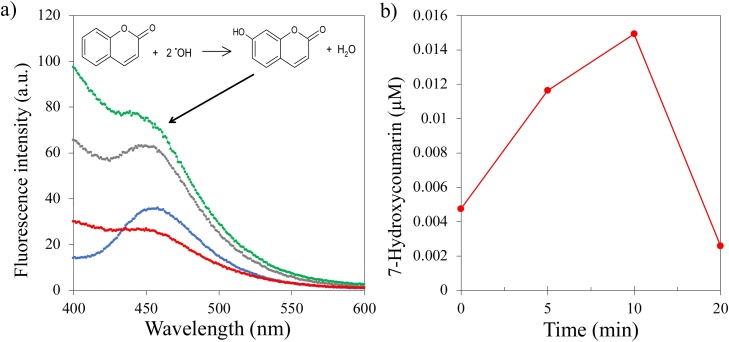
a) Fluorescence spectra of EF produced 7-HC at different times. Blue line: 0 min, gray line: 5 min, green line: 10 min, red line: 20 min of reaction. b) 7-HC concentration as a function of time. Experimental conditions: VAC, 2.5 g of Fe(II) loaded resin, 8.0 V, 30 mL min^−1^, pH 3, 0.4 mM coumarin in 0.05 M Na_2_SO_4_ at room temperature.

In order to find out how long this period needed to be, ^•^OH radical production was quantified by means of the fluorometric determination of 7-hydroxycumarin (7—HC) that results from the selective reaction of EF produced hydroxyl radical molecules with the coumarin probe as described by Medel et al. (2019) [39]. The resulting experimental data for the EF reactor under study is shown in Fig. 2 where the fluorescent response of 7—HC at 456 nm (after excitation at 332 nm) can be seen to increase during the first 10 min of polarization. After that period of time, the ^•^OH radical related response falls sharply, probably as a consequence of the accumulation and subsequent reduction of H_2_O_2_ at the cathode surface and the scavenging effect of Fe(III) [40,41].

From these experiments and from previous reports using the EF reactor in Fig. 1, the sequential adsorption/electrochemical operation scheme in which potential and flow direction are cycled, was defined (see Table 1).

### 3.2 Selection of operational parameters for the study of AC packed bed performance in an EF reactor

For this study, the selection of variables that influence the performance of the EF reactor depicted in Fig. 1, focused first on the source of AC particles that make up the packed bed in compartment C_2_. As can be seen in Table 2, two AC materials of different source were selected. In this regard, it is interesting to note that the surface of lignitic AC (LAC) as well as vegetal AC (VAC) is rich in oxygen-containing functional moieties, such as carboxylic acids, phenol, alcohol as well as aryl or alkyl ether moieties [3,42,43], and that this feature makes AC materials attractive for catalytic as well as for electrochemical applications [[43], [44], [45], [46]].

**Table 2:** Factor levels considered in the design of experiments.

Factor	Low Level (-)		High Level (+)	
(A) AC pretreatment	DIW (A-)		Acid (A+)	
(B) AC Source*	VAC (B-)		LAC (B+)	
(C) particle distribution**	Small sized particles (C-)	Medium sized particles (C0)		Mixed sized particles (C+)
(D) Amount of Fe(II) loaded resin (g)	0.25 (D-)		2.5 (D+)	

* Surface area values for VAC and LAC correspond to 1091 m^2^ g^−1^ and 548 m^2^ g^−1^, respectively.

** While (C+) corresponds to un-meshed VAC and LAC materials, (C-) corresponds to meshed samples for which the particle size window was 0.85 ≤ *x* ≤ 1 mm for VAC and 1.4 ≤ *x* ≤ 1.7 mm for LAC, respectively. (C0) samples correspond to meshed samples using a particle size window, *x*, of 1.0 ≤ *x* ≤ 1.18 mm for VAC and 2.0 ≤ *x* ≤ 2.36 mm for LAC, respectively.

In addition to source imposed differences, the activation of carbon substrates could change not only the material's morphology but also the chemical composition and oxidation degree of the surface [47]. In this regard, there are a variety of observations related to specific experimental treatments for AC. While Zhu et al. [48]. for example reported an increase of the surface area of about 25 times after activation of carbon with KOH and HCl, Yakout [49] reported the decrease of surface area due to the breaking of the material's pore walls and the expansion of micropores into meso and macropores during HNO_3_ erosion.

The acidic treatment of AC further modifies the surface characteristics of the adsorbent, such as the type and concentration of functional groups [10,42]. Fang et al. for example, reported an increase in the number of oxygen-containing functional groups (such as OH, HCO, R (CO) R’, COOH, and phenolic O-H) on the surface of AC upon HNO_3_ treatment. In this way, acid activation of AC results in a significant aliphatic AC oxidation that derives in oxygen containing functionalities on its surface [49]. Typically, carboxylic, ketonic and amine groups as well as other oxygen-containing moieties are expected to increase the polar and hydrophilic character of the surface; thus modifying adsorptive and electrocatalytic properties [10,42,49] that in turn, should influence the performance of AC materials in an EF reactor. In this way, in addition to the AC source, the application of an acid pre-treatment was defined a second variable for the study.

In the next stage and following a previous report in which it was determined that the optimum density of AC in the reactor shown in Fig. 1 corresponds to 0.52 g cm^−3^ [27], a third variable that is closely related to the specific surface area of AC, was selected in terms of the application of a mesh separation process to the LAC and VAC samples. As it is described in the experimental section and as can be observed in Table 2, three levels for each one of the two types of AC samples under study were defined for the particle separation process. It is interesting to note that as the mesh separation stage is applied to the AC samples, the resulting carbon packed bed will be characterized by a larger surface area (since the larger particles are removed) and a less packed structure (lower density) since the compartment volume C_2_ is fixed. As it will be discussed in an upcoming section, this difference will be relevant in terms of the electronic conductivity of the AC bed [27].

In addition to the AC surface area, morphology and chemical composition of the adsorbent, it is clear that the performance of the adsorbent/electrode material in the electro-Fenton reactor shown in Fig. 1, is strongly dependent on the amount of Fe(II) ions in solution. Since in this EF treatment system, Fe ions are provided by the cation loaded resin in compartment C_1_ during the adsorption stage (see Fig. 1c) [21,23,28,50], the amount of resin becomes another variable that was chosen to be considered. In Table 2, the four variables and their corresponding levels are shown.

### 3.3 Analysis of the color removal experimental results

As can be seen in Table 3, the multilevel DOE approach resulted in 24 runs that were carried out in random order in order to avoid experimental bias. Inspection of the corresponding data shows that the adsorption step that takes place during the first 20 min of the experiment, results in quite low color removal values; particularly in the case in which the high level of iron resin was employed (D+). This observation suggests that during the first minutes of the experiment and in the absence of electric polarization, Fe is competing with dye molecules for the adsorption sites of the AC substrate [[51], [52], [53], [54]]. The data in Table 3 also shows that as a consequence of the polarization stage that follows the first adsorption process, color removal increases dramatically, revealing that the electrochemical generation of the Fenton reagent in the vicinity of the AC-solution interphase gives rise to a fast an efficient dye degradation process.

**Table 3:** Experimental results of the DOE for CR% obtained from adsorption (after t = 20 min) and EF (after t = 40 min).

Run	A	B	C	D	AC Color removal, 20 min (%)	EF Color removal, 40 min (%)	Fe (mg L^−1^)
1	(-)	(-)	(-)	(-)	17.4 ± 0.41	98.4 ± 0.54	2.9 ± 0.16
2	(+)	(-)	(-)	(-)	11.6 ± 0.52	96.6 ± 0.47	4.0 ± 0.18
3	(-)	(+)	(-)	(-)	1.7 ± 0.5	75.7 ± 0.63	2.6 ± 0.16
4	(+)	(+)	(-)	(-)	7.6 ± 0.60	79.6 ± 0.47	5.6 ± 0.33
5	(-)	(-)	(0)	(-)	15.0 ± 0.65	92.8 ± 0.46	3.0 ± 0.16
6	(+)	(-)	(0)	(-)	0.6 ± 0.46	89.6 ± 0.33	5.2 ± 0.11
7	(-)	(+)	(0)	(-)	1.0 ± 0.60	49.0 ± 0.41	3.3 ± 0.12
8	(+)	(+)	(0)	(-)	3.2 ± 0.52	62.6 ± 0.34	5.7 ± 0.10
9	(-)	(-)	(+)	(-)	16.5 ± 0.51	98.2 ± 0.46	2.1 ± 0.31
10	(+)	(-)	(+)	(-)	5.2 ± 0.50	97.2 ± 0.62	4.5 ± 0.22
11	(-)	(+)	(+)	(-)	0.9 ± 0.30	96.6 ± 0.28	3.4 ± 0.26
12	(+)	(+)	(+)	(-)	4.3 ± 0.36	95.3 ± 0.54	6.0 ± 0.22
13	(-)	(-)	(-)	(+)	0.0 ± 0.14	95.2 ± 0.48	22.2 ± 0.42
14	(+)	(-)	(-)	(+)	0.0 ± 0.20	96.4 ± 0.48	36.0 ± 0.31
15	(-)	(+)	(-)	(+)	0.0 ± 0.13	89.5 ± 0.43	24.6 ± 0.31
16	(+)	(+)	(-)	(+)	0.0 ± 0.16	32.1 ± 0.34	30.6 ± 0.36
17	(-)	(-)	(0)	(+)	0.0 ± 0.21	94.7 ± 0.34	18.3 ± 0.29
18	(+)	(-)	(0)	(+)	0.0 ± 0.20	93.5 ± 0.38	31.9 ± 0.52
19	(-)	(+)	(0)	(+)	0.0 ± 0.25	74.3 ± 0.39	28.1 ± 0.30
20	(+)	(+)	(0)	(+)	0.0 ± 0.14	49.4 ± 0.62	20.4 ± 0.50
21	(-)	(-)	(+)	(+)	0.0 ± 0.24	95.0 ± 0.55	24.8 ± 0.26
22	(+)	(-)	(+)	(+)	0.0 ± 0.16	94.4 ± 0.50	30.8 ± 0.28
23	(-)	(+)	(+)	(+)	0.6 ± 0.61	96.1 ± 0.48	20.1 ± 0.29
24	(+)	(+)	(+)	(+)	1.2 ± 0.46	96.4 ± 0.45	37.0 ± 0.41

Data of the color removal percentage after 40 min of treatment in Table 3, also shows that the worst performing systems correspond to runs 16, 7 and 20 (32, 49 and 49 % color removal, respectively) and that the common feature of these three runs is that the source of the AC substrates is lignite (B+). As it was pointed out in the experimental section, LAC is characterized by roughly half the normalized surface of VAC (548 m^2^ g^−1^ for LAC *vs* 1091 m^2^ g^−1^ for VAC), so that its poorer performance when compared to VAC (which in all cases give rise to CR% values above 89.6 %), is consistent with the experimental results.

Analysis of the set of variables for runs performing with CR% values below 80 % (runs 3,4,7,8,16, 19 and 20), not only supports the fact that in all these cases LAC was used (B+), but also reveals that neither pre-treatment (A) nor the amount of Fe loaded resin (D) are a differentiating factor for explaining the poor performing response of these combinations (for the 7 worst performing runs, 4 are characterized by (A+) and another 4 by (D-)). Analysis of these 7 combinations in terms of the three mesh separation levels ((C+), (C0) and (C-)), shows that 3 experiments contained the smallest sized AC particles (C-), 4 runs were made with the intermediate particle size (C0) and surprisingly, there were no poor performing experiments in this group carried out with unmeshed AC samples (C+). At a first sight, this observation is unexpected since the smaller the particle size of the AC sample filling up compartment 2, the larger is the adsorbent and electrode surface area of the carbon material and therefore the higher should be the value of the CR%. The fact that small AC particle in compartment C_2_ results in low performing EF CR% values can however, be explained in terms of inter-particle contact and electrical conductivity as described by Zárate et al. (2018) [27] who reported that the density of AC in the reactor is an important factor for EF performance in a polarized AC packed bed. In this way, meshed AC samples (C-) and (C0) could be suffering from inefficient electrical contact between AC particles, thus resulting in a limited EF performance effect.

The pH and conductivity values of all the experiments performed were measured in the receiving tank shown in Fig. 1a (data not shown). Since these values remained basically unchanged for most of the experimental combinations explored in the DOE strategy (conductivity and pH values of 9 ± 1.27 mS cm^−1^ and 3 ± 0.30, respectively), it can be assumed that they do not interfere with the analysis of the effect of the parameters under study on the experimental CR% values.

**Fig. 3:**
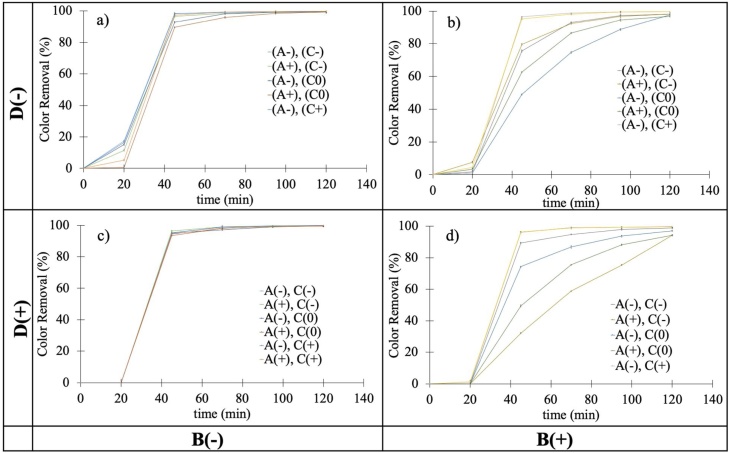
CR% *vs* time data for: a) VAC, and b) LAC, using 0.25 g of Fe loaded cation exchange resin (D-) and for, c) VAC, and d) LAC, using 2.5 g of Fe loaded cation exchange resin (D+). Error bars are not drawn since in all cases are smaller than 5 %.

In order to complement the data of Table 3, the color removal behavior of the different experiments over the four cycles that the experiment comprises is shown in Fig. 3. The information is arranged in four quadrants showing along a vertical axis the amount of Fe loaded resin (D) and along the horizontal direction, the AC source (B). The rest of the variables under study (A and C) are noted in the inset legend in each quadrant. Inspection of the relevant information in Fig. 3 shows that in all cases CR% reaches almost 100 % at long times and that the corresponding kinetics is substantially faster for VAC (Fig. 3a and c) than for LAC (Fig. 3b and d). As it was pointed out before, this difference is probably due to the larger specific area for VAC when compared to that of LAC. Comparison of the two top figures (Fig. 3a and b) with the two bottom ones (Fig. 3c and d) on the other hand, also shows that the amount of Fe in the system is an important factor and that in general terms, the use of the low level quantity of Fe loaded resin (D-) gives rise to faster CR% performance than employing a larger amount of iron in the experiment (D+).

Inspection of the two left side quadrants of Fig. 3 also shows that the analysis of the influence of A and C on the corresponding CR% data for VAC is quite difficult because the process is fast and highly effective. For experiments carried out using LAC on the other hand, some differences can be observed and the best performing experiments for the two levels of iron resin explored ((D+) and (D-)) can be seen to correspond to un-meshed AC samples (C+); a fact that was previously explained in terms of an improved interparticle electrical contact in AC samples characterized by a wider particle size distribution. The influence of the other variables could not be identified from inspection of the data in Fig. 3 because a given level of a specific variable ((A+) for example) sometimes gives rise to good and sometimes to poor CR% values.

Since there is a complex relationship between the experimental variables under study and in order to gain a deeper understanding of the relevant effects and interactions for the EF system under study, a statistical analysis of the DOE was carried out.

From the ANOVA that is shown Table 4, it can be clearly seen that the most significant effect in the process is exerted by factor B which, as it has been already pointed out, corresponds to the AC source.

**Table 4:** ANOVA Results for Color Removal experiments.

Item	Quadratic sum	DF	Mean square	*F-Test*	*P-Value*
A: AC Pre-treatment	4.09	1	4.09165	0.04	0.8553
B: AC Type	905.99	1	905.996	7.80	0.0209
C: AC Particle size	65.90	1	65.8971	0.57	0.4705
D: Amount of cation exchange resin	15.56	1	15.5599	0.13	0.7228
AB	3.45	1	3.44641	0.03	0.8670
AC	62.83	1	62.832	0.54	0.4807
AD	24.83	1	24.8327	0.21	0.6548
BC	147.46	1	147.461	1.27	0.2890
BD	76.77	1	76.7723	0.66	0.4372
CD	113.21	1	113.21	0.97	0.3493
ABC	77.22	1	77.2161	0.66	0.4359
ABD	448.50	1	448.503	3.86	0.0810
ACD	203.36	1	203.363	1.75	0.2184
BCD	29.30	1	29.2969	0.25	0.6275
Total Error	1045.24	9	116.137		
Total	7907.65	23			

*DF: Degrees of freedom.

In this way, the info in Table 4 shows that B is the only factor with statistical significance when compared to A, C and D by virtue of a P*-value* smaller than 0.05; a fact that reveals that only for B, the mean square value is smaller than a standardized 5 % estimate of experimental error. In addition, it is important to point out that the data in Table 4 also shows that second and third order interaction parameters are characterized by high P-*values*; thus suggesting the absence of statistically relevant effects of coupled variables.

**Fig. 4:**
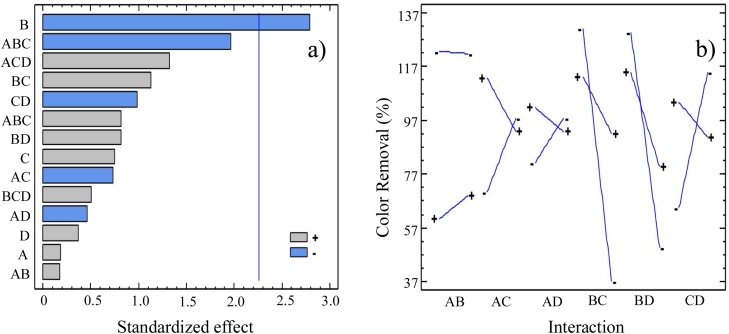
(a) Pareto diagram and (b) variable pair interaction map for CR% of the EF reactor under study.

The corresponding Pareto diagram in Fig. 4a also shows that B is the most influential factor since its bar is the only one characterized by a standardized effect larger than 2.25, (see blue line that represents the critical value). Fig. 4b on the other hand, also shows a diagram of the factor interaction effects on the CR% response; a map for which the lines with the largest slopes, represent the most influential factor interaction pairs.

Inspection of this figure shows that as anticipated, the interaction of the AC source, B, with either the mesh separation process (C) or using a different amount of cation exchange resin (D), constitutes an important effect on the CR% values of the EF process under study. In terms of the acid pre-treatment step (A) which, as has been mentioned, is relevant for the oxidized group content of the AC surface, Fig. 4b shows that while the interaction of A with the AC source, B seems to be irrelevant for the CR% result, the interaction of A with the particle size distribution defined by C, seems to have a moderate effect. This observation supports the inter-particle contact and derived electric conductivity effect that has been previously suggested for C and that should depend up to a certain point on A, since the pre-treatment oxidation process influences the hydrophilicity as well as the chemical composition of the surface of the AC material [27,[55], [56], [57], [58]].

Based on all the DOE experimental results after 120 min runs, an empirical equation was obtained using the software described in the experimental section. In this way, Eq. (4) predicts the value of the color removal percentage (CR%, response variable) as a function of A, B, C, D and their interactions for the EF reactor under study. As can be observed in the resulting equation and consistent with the experimental curves, the Pareto diagram and the variable interaction map, not only is the most important parameter for the CR% value, but it can also be seen that A is not important by itself but becomes moderately relevant when coupled to C (AC Particle size parameter).

(4) $$CR\left(\%\right)=93.41\;+1.94A-28.82B+9.30C+3.78D+2.40AB-11.89CA-6.43AD+18.21BC+11.31BD-15.96CD+15.22ABC-34.58ABD+24.7ACD+9.37BCD$$

Eq. (4) also confirms that the best conditions for the EF process under study correspond to the set (A-), (B-), (C+) and (D-) and that in fact, there is relatively important influence of D through its interaction with other parameters (see also Fig. 4b). In this context, and since D corresponds to the amount of cation exchange resin employed in the reactor in Fig. 1, we realized that D is a factor that is essentially independent of the AC substrate packed in C_2_ and that instead, is related to the reactor operation dynamics. Therefore, we decided to carry out additional experiments in order to understand how the different levels of D affect the presence of Fe ions in the vicinity of the polarized AC surface and in this context, how the distribution of Fe in the system affects the performance of the electrochemical generation of the Fenton reagent inside the reactor under study.

### 3.4 Analysis of the distribution of Fe inside of the EF reactor

In order to promote the Fenton electrochemical generation of ^•^OH radials inside compartment C_2_, it is necessary not only to use a suitable amount of Fe loaded cation exchange resin (D) in the reactor but also to make sure that the polarization and flow direction switching frequency is correct at the flow rate employed (see Fig. 1). In order to confirm the flowrate, switching time period (frequency) and properly asses the effect of the two levels of the amount of cation resin studied (D+) and (D-), Fe-transport experiments were carried out for the system under study.

**Fig. 5:**
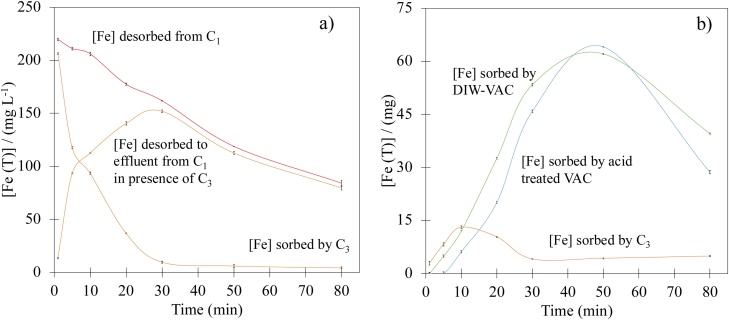
Iron *vs* time curves from experiments using different compartment arrangements with the EF reactor shown in Fig. 1.

The first experiment consisted in measuring the total amount of ionic Fe species that 1 g of a cation loaded resin in the C_1_ compartment releases as a function of time using a dye-free electrolyte at pH 3 flowing at 30 mL min^−1^. The red line with circle markers in Fig. 5a shows that in the absence of materials in C_2_ and C_3_, the concentration of Fe at the reactor’s outlet is the highest at the beginning of the experiment, falling in a linear fashion as time progresses.

The same experiment using an empty C_2_ compartment and 1 g of a Na^+^ saturated resin in C_3_, results in the curve with orange circle markers shown in Fig. 5a. The sort of volcano response observed, reflects the Fe retaining effect of the resin in C_3_ during the first 30 min of the experiment. After this time, the concentration of Fe in the effluent is similar to that of the first experiment, reflecting that the cation retention effect of the resin in C_3_ has faded away. The difference between these two curves can be seen in the cross marked curve in Fig. 5a and its integration on time using the flow rate (30 mL min^−1^), gives rise to the amount of Fe that is adsorbed on the resin contained in C_3_ at any given time. The integrated curve for the quantity of iron retained within the reactor is presented in the circle marked orange curve in Fig. 5b. Along with this volcano-type curve, Fig. 5b shows similar curves constructed using data in which the reactors were loaded with the two types of VAC (previously treated, (A+) and non-treated, (A-) with acid) in compartment C_2_. Comparison of the three curves reveals not only that the AC packed bed retains a substantially larger amount of ionic Fe in the reactor than the resin in C_3_ does, but also that this retention increases the residence time of the Fe species within the system. In this regard, it is important to point out that the 15 min long alternating flow direction stages that were used in the CR studies (see Table 1), guarantee that the Fe species are efficiently retained within the reactor; thus explaining the high dye decolorization efficiencies observed for all the experiments performed.

Comparison of the green and blue curves in Fig. 5b also reveal that acid pre-treatment has a modest effect in the iron retention capabilities of the VAC substrate. In this way, acid treated AC must have a larger amount of oxygen containing groups that, under acidic conditions, result in preferentially protonated AC surface groups that are uncapable of association with Fe cation molecules [26]. In order to find out if this slight effect could also be taking place in the CR experiments previously discussed, the concentration of Fe species was measured for all the combinations surveyed in the dye degradation experiments carried out with using the DOE approach.

**Fig. 6:**
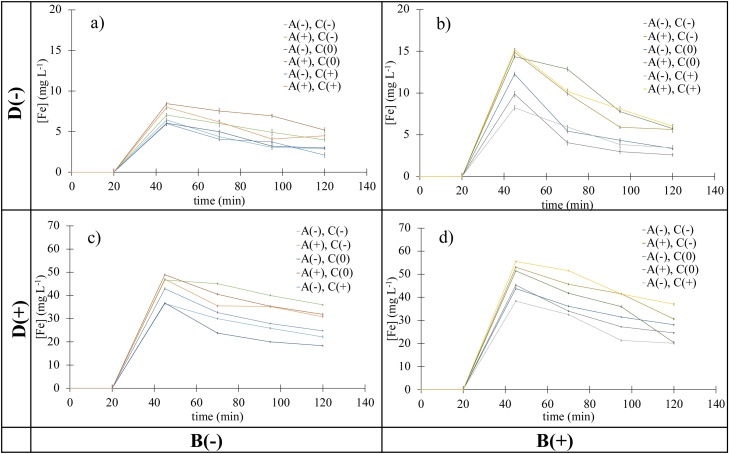
Total Fe concentration *vs* time data for: a) VAC, and b) LAC, using 0.25 g of Fe loaded cation exchange resin (D-) and for, c) VAC, and d) LAC, using 2.5 g of Fe loaded cation exchange resin (D+).

Fig. 6 shows the corresponding data where it can be seen that there are slightly smaller concentrations of released iron at long times for non-acid treated samples of the carbon substrate (A-); particularly for the case of LAC where the effect of the different variables under study is clearer (see also the corresponding data in Table 3). The acid treated AC samples on the other hand (A+) are characterized by larger concentrations of released Fe, reflecting limited retention properties due to the larger amount of protonated oxygen rich surface groups in the AC surface.

Inspection of Fig. 6 also shows that in all cases, Fe is released following a time dependence response similar to that observed in the orange curve of Fig. 5a. Since VAC has larger surface area than that of LAC, the amount of Fe released is in general terms smaller for the former than for the later. It is also interesting to note that although the amount Fe loaded resin for (D+) is 10 times larger than that for (D-), the data in Fig. 6 shows that the corresponding difference in concentration of released iron is only about 6 times (see also data in Table 3).

This observation suggests a sort of Fe saturation effect that is consistent with the higher CR% values obtained when a lower amount of Fe loaded resin (D-) was employed. In this regard, it is important to point out that this observation is supported by studies which found that an excessive amount of Fe ions close to the AC surface, results in an ^•^OH radical scavenger effect that seriously limits the efficiency of the EF degradation processes [59].

## 4 Conclusions

Using a model-dye pollutant solution and the corresponding temporal evolution of the color removal (CR) data, it was observed that the kinetics of AC adsorption can be substantially increased by coupling an EF process which in turn, can be triggered by electric polarization of the adsorbent. In this way, it was possible to conclude that a properly polarized AC packed bed, simultaneously works as an adsorbent as well as a 3D-electrode in an EF reactor.

A study on the effect of AC-pretreatment, -source, -particle size distribution and the amount of Fe(II) loaded resin, on the performance of the EF reactor, also showed that the most relevant parameter for color abatement kinetics is the AC source; suggesting a close relationship with the specific area of the carbonaceous material.

As expected, a complex relationship between the other experimental variables under study was found and modest effects and interactions were observed for the mesh separation as well as for the acid pre-treatment processes. In this way, while a wide particle size distribution for AC seems to favor inter-particle electrical contact, the absence of acid pre-treatment promotes less oxidized surface groups susceptible to protonation and therefore, larger Fe adsorption on the carbon surface. These two factors were found to improve the performance of the EF degradation process.

The amount of Fe loaded cation exchange resin in the reactor was also studied and the analysis of the corresponding data showed not only that the smaller amount of resin promotes a better CR performance, but also that there is an important amount of Fe adsorbed on the AC surface. Transport experiments revealed that in fact, the Fe retention properties of the system rely more importantly on the AC substrate than in the cation exchange resins. This finding suggests that most of the cationic Fe in the system remains all the time within the reactor and supports the high dye-discoloration efficiencies that were observed for EF experiments using cycling adsorption/polarization stages.

It is hoped that the conclusion reached from this work will contribute to the understanding of the performance of EF systems in which AC packed beds simultaneously work as adsorbent materials and as 3D-type electrodes.

## Declaration of Competing Interest

The authors declare that they have no known competing financial interests or personal relationships that could have appeared to influence the work reported in this paper.
